# SHORT SLEEP AND INSOMNIA ARE ASSOCIATED WITH ACCELERATED EPIGENETIC AGE

**DOI:** 10.1093/geroni/igac059.1436

**Published:** 2022-12-20

**Authors:** Cynthia Kusters, Eric Klopack, Eileen Crimmins, Teresa Seeman, Steve Cole, Judith Carroll

**Affiliations:** University of California, Los Angeles, Los Angeles, California, United States; University of Southern California, Leonard Davis School of Gerontology, Los Angeles, California, United States; University of Southern California, Los Angeles, California, United States; University of California Los Angeles, Los Angeles, California, United States; University of California Los Angeles, Los Angeles, California, United States; University of California, Los Angeles, Los Angeles, California, United States

## Abstract

Short sleep (<6 hours) and insomnia are independently associated with greater risk for age-related disease suggesting that insufficient sleep may accelerate biological aging. Epigenetic age acceleration is an estimate of biological aging that predicts morbidity and mortality. We tested whether insomnia symptoms and short sleep duration relates to epigenetic age in 2783 participants in the Health and Retirement Study. Insomnia and short sleep were associated with an 0.70(95%CI:0.23-1.17;P: 0.005) and 1.45(95%CI:0.67-2.24;P:0.001) years acceleration of GrimAge, respectively, as well as a faster pace of aging (DunedinPoAm; 0.015(95%CI: 0.005-0.024; P:0.006); 0.021(95%CI: 0.006-0.037; P:0.009)). Compared to healthy sleepers, Individuals with the combination of short sleep and insomnia had an accelerated GrimAge (1.34;95%CI: 0.49-02.19; P:0.003) and a greater DunedinPoAm (0.025; 95%CI: 0.009-0.041; P:0.004). Our findings indicate short sleep and insomnia are linked to epigenetic age acceleration, suggesting that these individuals have an older biological age that may contribute to risk for comorbidity and mortality.

